# Negative Transpulmonary Pressure Disrupts Airway Morphogenesis by Suppressing Fgf10

**DOI:** 10.3389/fcell.2021.725785

**Published:** 2021-12-01

**Authors:** Alice E. Stanton, Katharine Goodwin, Aswin Sundarakrishnan, Jacob M. Jaslove, Jason P. Gleghorn, Amira L. Pavlovich, Celeste M. Nelson

**Affiliations:** ^1^ Department of Chemical & Biological Engineering, Princeton University, Princeton, NJ, United States; ^2^ Lewis Sigler Institute for Integrative Genomics, Princeton University, Princeton, NJ, United States; ^3^ Department of Molecular Biology, Princeton University, Princeton, NJ, United States

**Keywords:** tissue morphodynamics, lung liquid, mechanical stress, mechanotransduction, pulmonary hypoplasia, congenital diaphragmatic hernia (CDH), tracheal occlusion

## Abstract

Mechanical forces are increasingly recognized as important determinants of cell and tissue phenotype and also appear to play a critical role in organ development. During the fetal stages of lung morphogenesis, the pressure of the fluid within the lumen of the airways is higher than that within the chest cavity, resulting in a positive transpulmonary pressure. Several congenital defects decrease or reverse transpulmonary pressure across the developing airways and are associated with a reduced number of branches and a correspondingly underdeveloped lung that is insufficient for gas exchange after birth. The small size of the early pseudoglandular stage lung and its relative inaccessibility *in utero* have precluded experimental investigation of the effects of transpulmonary pressure on early branching morphogenesis. Here, we present a simple culture model to explore the effects of negative transpulmonary pressure on development of the embryonic airways. We found that negative transpulmonary pressure decreases branching, and that it does so in part by altering the expression of fibroblast growth factor 10 (*Fgf10*). The morphogenesis of lungs maintained under negative transpulmonary pressure can be rescued by supplementing the culture medium with exogenous FGF10. These data suggest that *Fgf10* expression is regulated by mechanical stress in the developing airways. Understanding the mechanical signaling pathways that connect transpulmonary pressure to FGF10 can lead to the establishment of novel non-surgical approaches for ameliorating congenital lung defects.

## Introduction

Branching morphogenesis is the recursive developmental program used to build ramified epithelia including the airways of the lung and the ducts of the mammary gland and kidney (Metzger and Krasnow, 1999; Affolter et al., 2003; Morrisey and Hogan, 2010; Varner and Nelson, 2014; Goodwin and Nelson, 2020). In the lungs of mammals, the branching process generates a network of thousands of airways that are used to conduct air for breathing after birth. In the mouse, the developing airway epithelium can form new branches laterally off a main stem (also known as domain branching or monopodial branching) or by splitting the parent stem into two equivalent daughter branches (also known as terminal or dichotomous bifurcation) (Metzger et al., 2008).

The initiation of branching in the murine lung is regulated in part by coordinated signaling between the developing airway epithelium and its surrounding mesenchyme, relying primarily on fibroblast growth factor 10 (FGF10) (Sekine et al., 1999; Ohuchi et al., 2000; Shannon and Hyatt, 2004; Jones et al., 2019). *Fgf10* is expressed in the subjacent mesenchyme and influences epithelial differentiation and morphogenesis by binding to its cognate receptor, fibroblast growth factor receptor 2IIIb (FGFR2IIIb), which is expressed in the epithelium (Bellusci et al., 1997; Park et al., 1998; De Moerlooze et al., 2000; Weaver et al., 2000; Danopoulos et al., 2019a). Mice with a partial loss of *Fgf10* develop poorly branched, hypoplastic lungs (Ramasamy et al., 2007), whereas those with a complete loss fail to form the airway epithelium (Min et al., 1998; Sekine et al., 1999; Ohuchi et al., 2000). Conversely, ectopic expression of *Fgf10* ubiquitously (Volckaert et al., 2013) or specifically in the airway epithelium (Nyeng et al., 2008) also impairs lung development, in this case by promoting epithelial proliferation, inhibiting epithelial differentiation (Volckaert et al., 2013), and causing the formation of aberrant smooth muscle in the mesenchyme (Nyeng et al., 2008).

Even though FGF10 is accepted as a major regulator of airway branching morphogenesis, the upstream stimuli and downstream targets of FGF10 signaling remain poorly understood. *Fgf10*-null embryos exhibit lung agenesis and conditional gene deletions are complicated by incomplete FGF10 inactivation due to sequestration in the extracellular matrix (Patel et al., 2007; Jones et al., 2019). Among the well-characterized signaling pathways downstream of FGF10 include the mitogen-activated protein kinase (MAPK) cascade, which is necessary for airway epithelial branching (Ramasamy et al., 2007; Liu et al., 2008; Abler et al., 2009; Tang et al., 2011; Yin and Ornitz, 2020). FGF10 signaling is regulated in part by an inducible inhibitor, Sprouty 2 (SPRY2), which is expressed in the distal tips of the branching epithelium in response to FGF10, thus forming a negative feedback loop (Mailleux et al., 2001; Tefft et al., 2002). The expression of *Fgf10* around new branch sites has been postulated to induce an increase in the expression of *Spry2* as the branches elongate (Warburton et al., 2005). Sprouty 4 (SPRY4) has redundant functions and, along with SPRY2, negatively regulates FGF10-induced MAPK signaling (Taniguchi et al., 2007). Another well-characterized signaling cascade is the negative feedback loop involving sonic hedgehog (SHH) and bone morphogenic protein 4 (BMP4) (Prince, 2018). Activated FGFR2 signals through the ETS-related transcription factors ETV4 and ETV5 and induces epithelial expression of *Shh*, which in turn negatively regulates *Fgf10* expression in the adjacent mesenchyme (Herriges et al., 2015). Computational models suggest that FGF10-SHH interaction alone is sufficient to promote much of the branching that occurs in the early lung (Cellière et al., 2012; Herriges and Morrisey, 2014). In contrast with predictions from culture experiments (Warburton et al., 2005), recent studies suggest that the spatial location of FGF10 may be less important than the actual level of its expression (Volckaert et al., 2013; Herriges and Morrisey, 2014).

In addition to these biochemical signals, the embryonic lung is exposed to a variety of mechanical forces (Nelson and Gleghorn, 2012; Hogan, 2018). As the lung develops, the epithelium secretes fluid into the lumen of the airways (Mccray et al., 1992b; a;Harding and Hooper, 1996; Olver et al., 2004). This luminal fluid creates a positive distending transpulmonary pressure that has been measured at the late stages of lung development to be on the order of 200–400 Pa (1.5–3 mm Hg) in several animal models (Adams et al., 1967; Liggins and Kitterman, 1981; Fewell and Johnson, 1983; Blewett et al., 1996; Harding and Hooper, 1996; Schittny et al., 2000; Olver et al., 2004). Maintenance of this positive transpulmonary pressure is required for normal branching of the distal epithelium (Alcorn et al., 1977; Adzick et al., 1984; Moessinger et al., 1990). Airway hypoplasia, or an underdeveloped lung, is associated with several conditions that affect the mechanical environment of the fetal chest cavity, including congenital diaphragmatic hernia (CDH, in which a weakened diaphragm permits herniation of abdominal organs into the thoracic cavity) and pleural effusion associated with or without fetal hydrops (in which excess fluid accumulates between the lungs and the chest cavity) (Kitagawa et al., 1971; Carroll, 1977). In both cases, the fetal lungs are compressed by either tissue or fluid, although it remains unclear whether the resulting hypoplasia is commonly due to effects from confinement, altered transpulmonary pressure, or both (Sherer et al., 1990). Severely hypoplastic lungs have fewer branch points, resulting in a reduced number of airways and a decreased surface area for gas exchange (Kitagawa et al., 1971). Tracheal occlusion increases transpulmonary pressure and reverses airway hypoplasia in animal models (Alcorn et al., 1977; Kitano et al., 2001; Davey et al., 2003; Maltais et al., 2003; Baird et al., 2008; Grushka et al., 2010; Beck et al., 2012; Sosa-Sosa et al., 2012), and chronic drainage of excess pleural fluid can rescue lung development in humans (Blott et al., 1988). Rodent models of CDH present with a reduction in the number of branches (Baird et al., 2008; Fox et al., 2018; Nguyen et al., 2019) and reduced expression of both *Spry2* (Friedmacher et al., 2013) and *Fgf10* (Teramoto et al., 2003), although it remains unclear whether these effects are due to changes in the mechanical environment surrounding the lungs, biochemical signaling from nitrofen induction of CDH, or both (Keijzer et al., 2000). Tracheal occlusion in the pseudoglandular murine lung results in increased levels of *Fgf10* and phospho-ERK and decreased levels of *Spry2*; consistently, tracheal occlusion of FGFR2B-null lungs does not induce an increase in branching (Unbekandt et al., 2008). Therefore, the FGF10-FGFR2-SPRY2 pathway is activated downstream of positive transpulmonary pressure (Unbekandt et al., 2008). However, it is unclear whether this same signaling cascade is affected by the magnitude or direction of pressure.

Here, we describe a simple culture system using murine embryonic lung explants to isolate the effects of negative transpulmonary pressures from those of physical confinement on early lung development. Using this system, we found that compressing the lungs under negative transpulmonary pressure disrupts branching of the airways without affecting basal epithelial area, as inferred from quantitative morphometric analysis. Negative transpulmonary pressure also decreases the expression of *Fgf10* and its inhibitor, *Spry2*, which suggests that the tissues of the developing lung can sense the direction of pressure. Exogenous addition of FGF10 rescues the branching defect of airways held under negative transpulmonary pressure, revealing that this growth factor acts as a critical transducer of mechanical signaling during early lung development.

## Materials and Methods

### Culture of Lungs *Ex Vivo*

**FIGURE 1:**
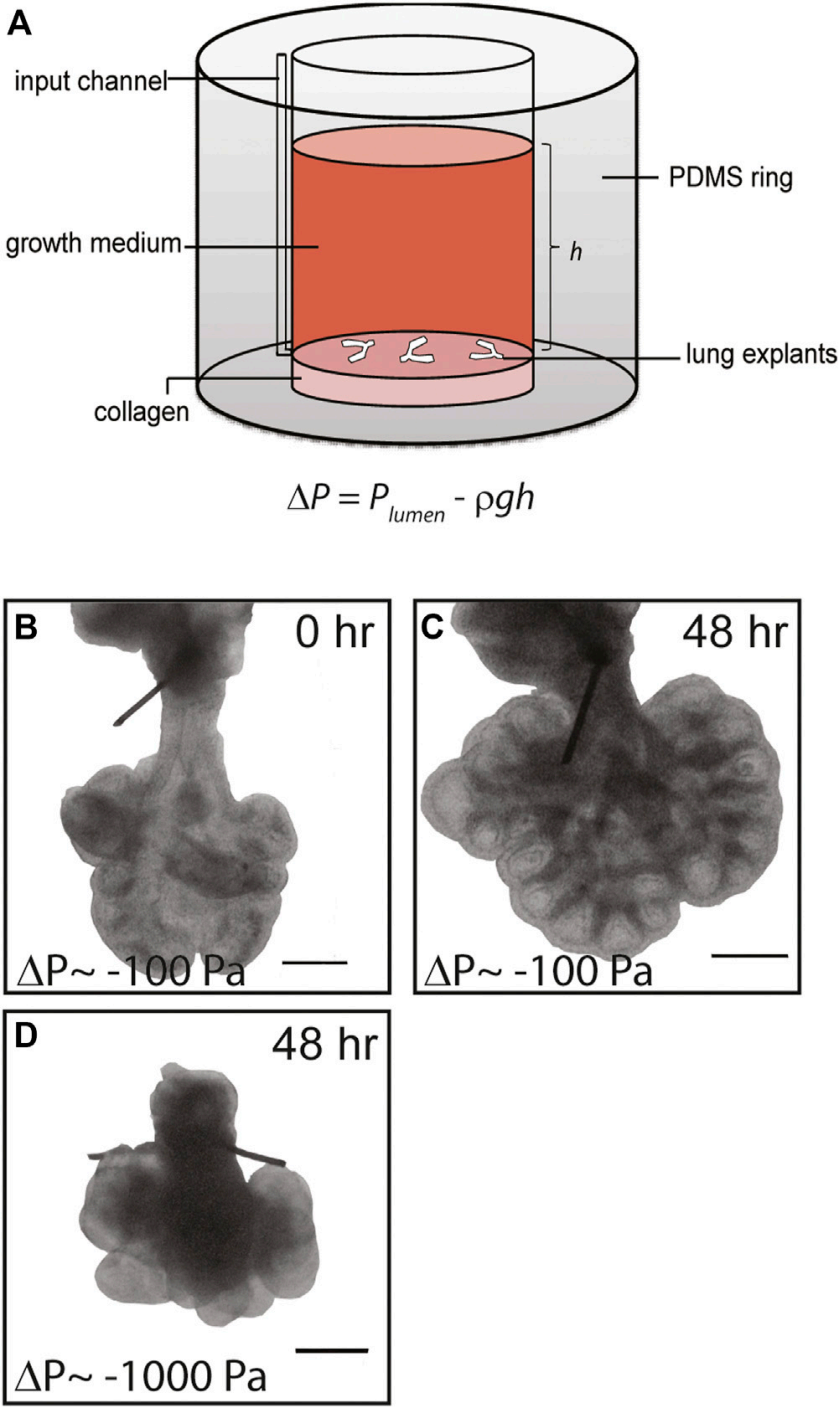
Simple culture system to apply negative transpulmonary pressure to embryonic lung explants *ex vivo.* **(A)** Schematic of culture model. Transpulmonary pressure, *ΔP*, is given as the difference between the pressure within the lumen of the explant, *P*_*lumen*_, and the hydrostatic pressure above the explant *P*_*reservoir*_, which is equal to *ρgh*. **(B**,**C)** Representative brightfield images of *E*12.5 lung explants **(B)** at the start of culture and **(C)** after 48 h at *ΔP* = −100 Pa. **(D)** Brightfield image of lung explant cultured for 48 h at *ΔP* = −1,000 Pa. Scale bars, 250 µm.

Embryonic lungs from timed-pregnant CD1 mice at embryonic day (*E*) 12.5 were micro-dissected in chilled phosphate-buffered saline (PBS) supplemented with penicillin/streptomycin (1:1,000; Invitrogen). The tracheae were occluded using a 10-0 suture (ARO Surgical) and the sutured lungs were placed in a culture system designed to control the hydrostatic pressure acting on the explants (Figure 1A). Briefly, three or four explanted lungs were placed on top of plasma-treated tissue culture-grade polystyrene (TCPS) or a thin layer of type I collagen (rat tail; BD Biosciences) polymerized within a ring of polydimethylsiloxane (PDMS). A hydrostatic pressure head was created by introducing culture medium into the PDMS ring; the culture medium was comprised of DMEM:F12 without HEPES (Fisher) supplemented with penicillin/streptomycin (1:1,000) and 0.5% fetal bovine serum (FBS; Atlanta Biologicals). Cultured explants were incubated at 37°C in 5% CO_2_ and optimal humidity under different volumes of culture medium; the height of this medium was used to modulate the hydrostatic pressure applied to the explants. The medium was refreshed every 24 h. In FGF10 supplementation experiments, 50 ng/ml of FGF10 (Sigma) was added to the culture medium and the cultures were maintained for 24 h.

To confirm tracheal closure, FluoSphere beads with diameter of 1 µm (Invitrogen, F8821) were vortexed and then diluted 1:100 in sterile PBS. Beads were then loaded into a pulled glass needle using a capillary loading pipette, and the needle was mounted on a Pneumatic PicoPump (World Precision Instruments). Dissected lungs were mounted on membranes at an air-liquid interface (to immobilize them prior to injection) either with open or sutured tracheae, and immediately injected with beads in both the left and right distal-most buds. Time-lapse imaging was performed immediately after injection, with all timelapses being recorded within 15 min of injection.

To investigate the effects of hypoxia on branching morphogenesis, explanted lungs were cultured for 24 h under either humidified normoxic conditions (95% air and 5% CO_2_) or in a modular incubator chamber (Billings-Rothenberg, Inc.) filled with hypoxic gas (94% N_2_, 1% O_2_, and 5% CO_2_). To culture lung explants under different magnitudes of transpulmonary pressure without altering the height or volume of the culture medium, an Arduino Uno microcontroller was used to regulate the pressure of culture medium that was applied using a syringe pump.

### Immunofluorescence Analysis

Freshly dissected *E*12.5 lungs, as well as those cultured *ex vivo*, were fixed in 4% paraformaldehyde and processed for staining. Fixed lungs were washed with PBS and blocked in buffer containing 10% goat serum supplemented with 0.1% Triton X-100 for 12 h at room temperature. The blocking buffer was removed and samples were incubated in primary antibody against E-cadherin (rat; Invitrogen #13-1900), alpha-smooth muscle actin (αSMA; mouse; Sigma #A5228), or pERK1/2 (rabbit; Cell Signaling #9101) diluted 1:100 in blocking buffer for 12 h at 4°C. The samples were washed extensively and then incubated in secondary antibody (Alexa-conjugated goat-anti-rat or goat-anti-rabbit; Invitrogen) diluted 1:1,000 in blocking buffer for 12 h at 4°C. Samples were washed again and stored in chilled PBS prior to imaging.

To enhance the clarity of imaging, the tracheal sutures were removed and the stained samples were cleared by submerging in isopropyl alcohol followed by Murray’s solution (1:2 ratio of benzyl alcohol: benzyl benzoate; Sigma) until the lungs became transparent, as described previously (Kim et al., 2013). Samples were imaged on a Nikon Ti-U inverted microscope with a Hamamatsu camera and a spinning disk confocal attachment (X-Light) using a Plan Apo 4× 0.2 NA, Plan Fluor 10× 0.3 NA, or S Plan Fluor 20× 0.45 NA objective. Confocal stacks were analyzed as described below. pERK intensity was measured manually in Fiji. Mean gray value was measured in three ROIs of 30 × 30 pixels (10.8 × 10.8 µm) per sample and then normalized to background intensity.

### Skeletonization and Quantification of Allometric and Isometric Growth

The three-dimensional (3D) morphology of the airways at each of the experimental pressures and timepoints were reconstructed from z-stacks of confocal images of lungs using the Amira^®^ software package. The branching architecture of the airways was recreated using a skeletonization algorithm to define the topological centroid at each position in the 3D geometry. Fold-change in number of branches and basal epithelial surface area were used to infer the relationship between allometric growth (branching) and isometric growth (basal epithelial area) as a function of time and transpulmonary pressure.

### Quantitative Real-Time Polymerase Chain Reaction

Following extensive washing in PBS and removal of the tracheal sutures, total RNA was isolated from 7 to 8 lung explants from each experimental group at the desired time points using an RNeasy Mini Kit (Qiagen). cDNA was reverse transcribed from 1 µg of purified RNA using the Verso cDNA synthesis kit (Thermo Scientific), and quantitative RT-PCR was performed using iQ SYBR Green Supermix and an iCycler Realtime PCR Detection System (BioRad). Primers (Table 1) were designed using Beacon Designer Software (Premier Biosoft) and determined to be specific by BLAST and dissociation curve analysis. The expression level of each mRNA was normalized to that of 18S in the same sample.

**TABLE 1:** Primer sequences used for quantitative RT-PCR.

Gene	Sequence
*Fgf10*	Fwd: CAA​CTC​CGA​TTT​CCA​CTG​ATG​T
	Rev: GCT​GTT​CTC​CTT​CAC​CAA​GT
*Spry2*	Fwd: TGC​ACA​TCG​CTG​GAA​GAA​GAG​GAT
	Rev: TCC​ATC​AGG​TCT​TGG​CAG​TGT​GTT
*Shh*	Fwd: GGA​AAA​CAC​TGG​AGC​AGA​CC
	Rev: CCA​CGG​AGT​TCT​CTG​CTT​TC
*Sftpc*	Fwd: TTG​TCG​TGG​TGA​TTG​TAG​GG
	Rev: TGG​AAA​AGG​TAG​CGA​TGG​TG
*Cftr*	Fwd: TTG​CAG​AAC​AAG​ACA​ACA​CAG​TTC
	Rev: AAA​GAA​ATC​CTT​GCA​CGC​TGA
*18S*	Fwd: TCA​GAT​ACC​GTC​GTA​GTT​C
	Rev: CCT​TTA​AGT​TTC​AGC​TTT​GC

### Modeling of Oxygen Diffusion

We modeled the oxygen concentration as a function of the height of the column of media by using Fick’s second law of diffusion:

$$\frac{\partial \varphi }{\partial t}=D\frac{\partial^{2}\varphi }{\partial x^{2}}$$ (1)

Where φ = oxygen concentration, t = time, x = distance within the media column starting from the air-liquid interface. The lowest oxygen concentrations will be reached at the bottom of the media column when the oxygen concentration profile reaches a steady state such that $\frac{\partial \varphi }{\partial t}=0$ , and Eq. 1 becomes

$$D\frac{\partial^{2}\varphi }{\partial x^{2}}=0$$ (2)

Integrating twice from x=0 (air-liquid interface) to $x=x_{b}$ (bottom of media column) gives

$$\varphi \left(x\right)=\varphi \left(0\right)+\frac{d\varphi \left(0\right)}{dx}x$$ (3)

At the air-liquid interface (x = 0), the oxygen concentration follows Henry’s Law such that

$$\varphi \left(0\right)=\frac{Po_{2}}{Kc}$$ (4)

Where Kc = Henry’s constant for oxygen in plasma and $Po_{2}$ = partial pressure of oxygen calculated as

$$Po_{2}=Fo_{2}\left(P_{ATM}-P_{H_{2}O}\right)$$ (5)

Where $Fo_{2}$ = fraction of oxygen in the atmosphere, $P_{ATM}$ = atmospheric pressure, $P_{H_{2}O}$ = vapor pressure of water. We assume the lung consumes oxygen at a constant rate, such that at $x_{b}$ the oxygen flux, J , is

$$J=\frac{r}{A}$$ (6)

Where r = rate at which the lung consumes oxygen and A = the area of the bottom surface of the vessel. From Fick’s first law

$$J=-D\frac{d\varphi }{dx}$$ (7)

Rearranges to

$$\frac{d\varphi }{dx}=-\frac{r}{DA}$$ (8)

Using the oxygen concentration at x = 0 and $\frac{d\varphi }{dx}$ at x = $x_{b}$ as boundary conditions for Eq. 3 gives

$$\varphi =\frac{Po_{2}}{K_{c}}-\frac{xr}{DA}$$ (9)

In a Python script, we used Eqs. 4, 5, and 9 to calculate the concentration of O_2_ at the bottom of columns of media from 0 to 100 mm tall, using the values of constants reported in Table 2. We estimated the rate of oxygen consumption, r , by one lung based on literature values for the consumption of oxygen by tissues and from the volume of an *E*11.5 lung cultured for 48 h as measured by confocal imaging. To account for the possibility of multiple lungs being cultured together in a well and to investigate the sensitivity of our calculations to the rate of oxygen consumption by the tissue, we repeated this calculation for r values up to forty times higher than our estimated r .

**TABLE 2:** Constants used for modeling of oxygen diffusion.

Constant	Value	References
$K_{c}$	1/0.0031 mmHg dL/ml (of O_2_ in plasma)	West (2012)
$P_{H_{2}O}$	47.067 mmHg (at 37°C)	West (2012)
$P_{ATM}$	760 mmHg	West (2012)
$F_{O_{2}}$	21%	West (2012)
r	0.023 ml O_2_/ml tissue/min × 1 × 10^–5^ ml lung = 0.023 × 10^–5^ ml O_2_/min	Trowell (1959)
D	2.76 × 10^–5^ cm^2^/s (of O_2_ in water)	Yoshida and Ohshima (1966)
A	9.6 cm^2^ (one well of a 6-well plate)	ThermoFisher Scientific (2020)

### Statistical Analysis

Data are reported as mean and error bars denote standard error of the mean. All data are from three experimental replicates conducted on different days, unless otherwise noted. Comparisons between two experimental groups were performed using two-tailed unpaired *t*-tests. Comparisons between multiple groups were performed using one- or two-way analysis of variance (ANOVA) with Tukey post-hoc analysis. Statistically significant differences are indicated as *p < 0.05, **p < 0.01, ***p < 0.001, ****p < 0.0001.

## Results

### Culture System to Generate Negative Transpulmonary Pressure Across Embryonic Lung Explants

Compression from physical confinement or excess fluid around the lungs have separately been implicated in several fetal anatomic disorders that result in pulmonary hypoplasia (Kitterman, 1988), but the underlying molecular mechanisms that connect alterations in transpulmonary pressure to abnormal lung development are unknown. To address this gap and to define whether fluid pressure can affect the earliest stages of lung development, which are inaccessible to experimental manipulation *in vivo*, we created a simple system to modulate the transpulmonary pressure across embryonic lung explants cultured *ex vivo* (Figure 1A). Lungs dissected from mice at *E*12.5 were sutured to occlude the tracheae (Supplementary Movies S1, S2) and the sutured lungs were anchored on a thin layer of type I collagen or plasma-treated tissue culture-grade polystyrene (TCPS). Culture medium was added to the reservoir to provide a hydrostatic head of height *h*, which generated a hydrostatic pressure of *P*_*reservoir*_ *= ρgh*. The transpulmonary pressure experienced by the developing airway epithelium was thus taken to be *ΔP = P*_*lumen*_ *– P*_*reservoir*_. At the start of the experiment, following suturing of the trachea, we assumed *P*_*lumen*_ to be 0 Pa above atmospheric.

Lung explants were cultured in this system and monitored for 24–72 h. Brightfield microscopy analysis revealed that the extent of branching and overall size of the lungs increased with time in culture (Figures 1B,C), consistent with previous studies of branching in embryonic lung explants with or without tracheal occlusion (Laberge and Flageole, 2007; Baird et al., 2008; Unbekandt et al., 2008; Grushka et al., 2010). The final morphology of the lungs depended on the hydrostatic pressure introduced to the reservoir: higher hydrostatic pressures, which corresponded to larger negative transpulmonary pressures, led to a marked reduction in the overall size of the lungs and stunted airway branching morphogenesis (compare Figures 1C,D). These data suggest that negative transpulmonary pressure disrupts airway morphogenesis in culture, consistent with the hypoplastic branching phenotypes observed at later stages of lung development in animal models and in the clinic.

### Negative Transpulmonary Pressure Reduces Allometric Growth (Branching of the Airways) Without Affecting Isometric Growth (Basal Epithelial Surface Area)

**FIGURE 2:**
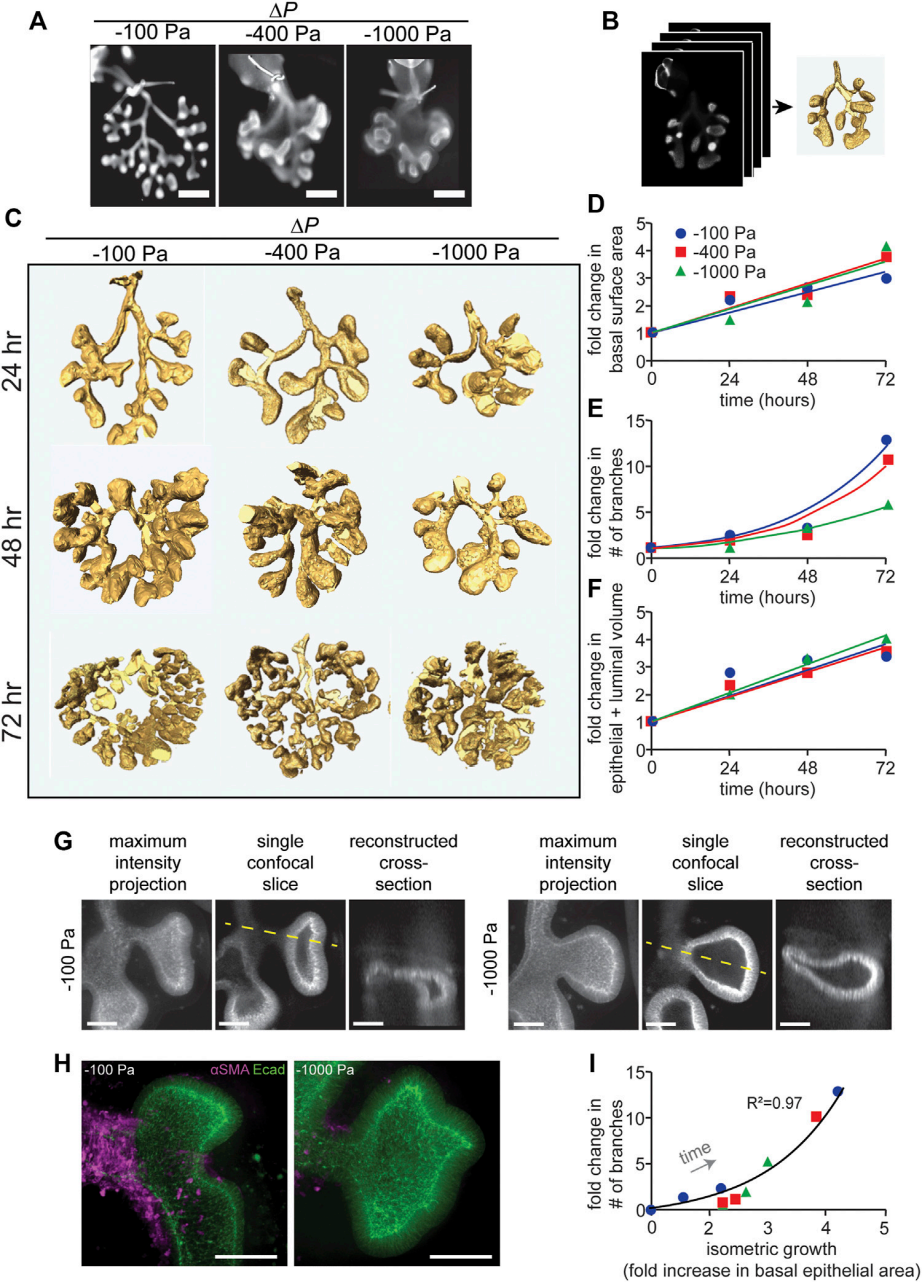
Quantitative morphometric analysis of the effects of negative transpulmonary pressure on airway branching. **(A)** Immunofluorescence analysis for E-cadherin in lung explants that were maintained for 72 h at the indicated transpulmonary pressures. The trachea (with occluding suture) is evident at the top of each image. Scale bars, 250 µm. **(B)** Schematic of procedure to obtain 3D volume renderings of the airways. Confocal stacks of E-cadherin-stained lungs were used to generate 3D solid models of the airways. **(C)** 3D volume renderings of lungs maintained at *ΔP* of −100, −400, and −1,000 Pa for 24, 48, and 72 h. Renderings are to scale. **(D**–**F)** Graphs of fold-change in **(D)** the basal epithelial surface area of the airways, **(E)** the number of branches, and **(F)** the volume of the airways (inclusive of the lumen) as a function of time and transpulmonary pressure. **(G)** Maximum intensity projections, single confocal slices, and reconstructed cross sections (taken along the dashed yellow line) of explants held under *ΔP* of −100 or −1,000 Pa and stained for E-cadherin. **(H)** Immunofluorescence analysis for E-cadherin (green) and αSMA (magenta) in lung explants that were maintained for 24 h at the indicated transpulmonary pressure. Scale bars, 50 µm. **(I)** Relationship between allometric growth (branching) and isometric growth (basal epithelial surface area) as a function of time and transpulmonary pressure. Points indicate experimental measurements; lines indicate linear and exponential curve fits to the data (N = 3 experimental replicates).

To characterize the time-dependent effects of transpulmonary pressure on the architecture of the developing airways, lung explants were removed from culture at 24-h intervals. We used immunofluorescence analysis for E-cadherin to label the airway epithelium (Figure 2A) and reconstructed the three-dimensional (3D) architecture of the airways from confocal image stacks, which we represented as volume renderings (Figures 2B,C). Even at early timepoints, increasingly negative transpulmonary pressures appeared to result in a reduction in the number of branches.

To quantify the morphogenesis of the lung explants, we used the 3D interactive volume renderings to measure the basal epithelial surface area and epithelial volume (inclusive of the lumen) and to count the number of branches as a function of time and transpulmonary pressure. Fold-increases in these parameters were calculated relative to explants immediately after dissection, prior to culture or tracheal occlusion. Consistent with our qualitative observations, we found that the number of branches increased as a function of time, and that the rate of branching decreased with increasingly negative transpulmonary pressure (Figure 2E). In contrast, both the basal epithelial surface area (Figure 2D) and epithelial + luminal volume (Figure 2F) increased linearly with time, surprisingly with no discernable change as a function of transpulmonary pressure. As negative pressure increased, the terminal lung buds became more cyst-like in morphology (Figure 2G), with branches that lacked smooth muscle coverage (Figure 2H) and rounded rather than branched terminal ends.

To compare the branching patterns between pressure conditions quantitatively, we took advantage of an allometric analysis framework created previously to describe the time-dependent architecture of the avian lung (Gleghorn et al., 2012). Allometric analysis provides a scaling law, of the form *y α x* ^*a*^, to describe the relationship between two variables of interest (Huxley, 1924; Huxley and Tessier, 1936). We used such an analysis to define the relationship between branching (or allometric growth) and basal epithelial area (or isometric growth). We found that data from individual explants that were tracked over time collapsed onto a single curve (Figure 2I), which was well described by a power law with scaling coefficient, *a* = 1.67 (*R* ^2^ = 0.97). Consistent with our quantitative morphometric analysis, data from explants held under all transpulmonary pressures collapsed onto the same curve. Altogether, these data suggest that negative transpulmonary pressure significantly affects the rate of branching but not the rate of isometric growth (change in the basal surface area) of the airway epithelium in mouse embryonic lungs cultured *ex vivo*.

**FIGURE 3:**
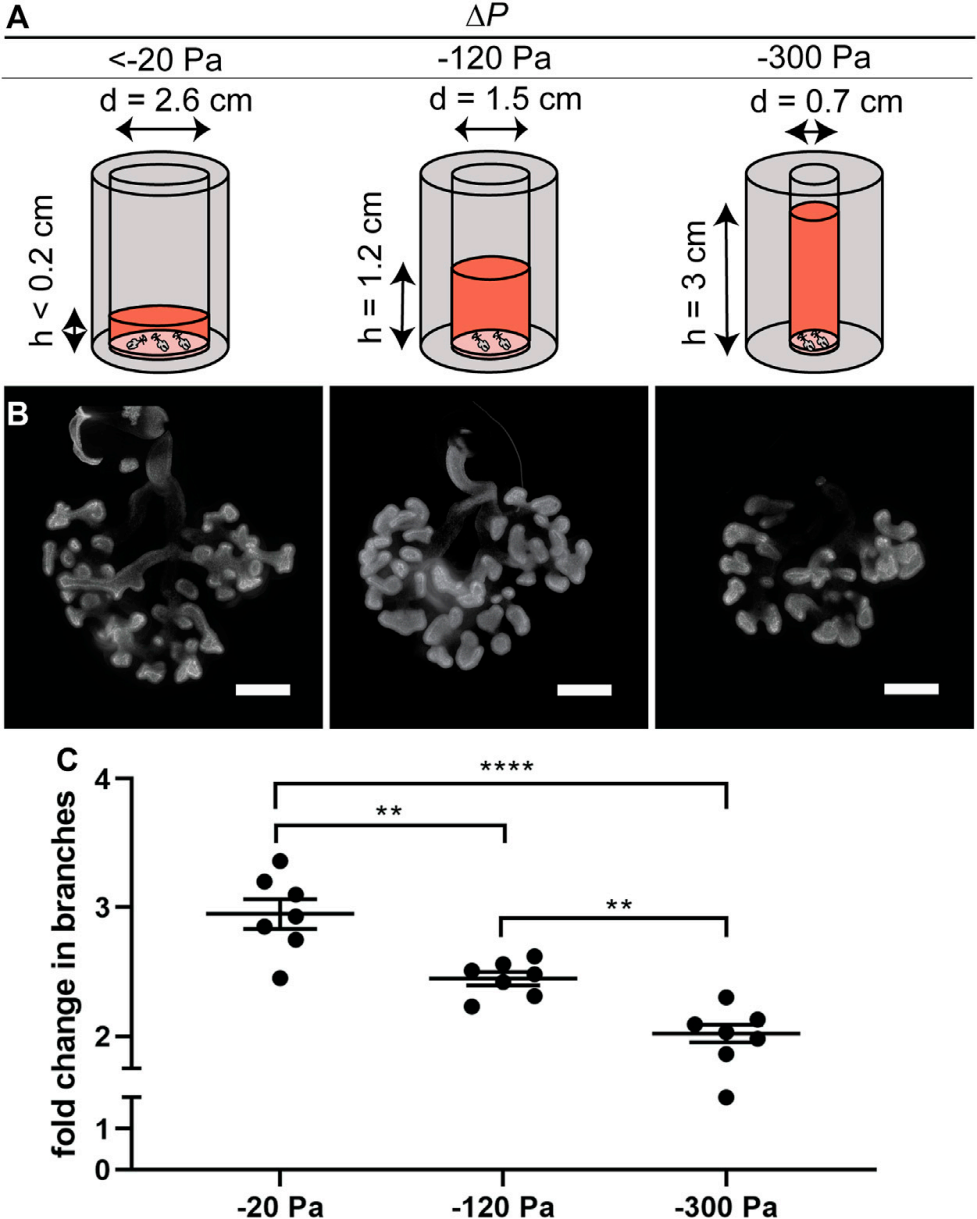
Effect of negative transpulmonary pressure on branching of explants held under isovolumetric conditions. **(A)** Schematic of culture setup for isovolumetric negative transpulmonary pressure. **(B)** Immunofluorescence analysis for E-cadherin in lung explants cultured for 48 h under indicated transpulmonary pressures. **(C)** Fold change in number of branches after culture for 48 h under the indicated transpulmonary pressures (N = 3 experimental replicates; 7 explants/group). Scale bars, 200 µm.

To determine whether the effects on branching were due to the height (pressure) of the column of culture medium or its volume, we carried out a second series of experiments in which the volume was held constant while the pressure was varied. We achieved constant volume (isovolumetric) application of pressure by varying the diameter of the culture vessel (Figure 3A). These experiments yielded similar results to those described above (Figures 3B,C), confirming our observations that negative transpulmonary pressure leads to a decrease in the number of terminal branches.

### Negative Transpulmonary Pressure Decreases Branching Independently of Effects on Oxygen Transport

**FIGURE 4:**
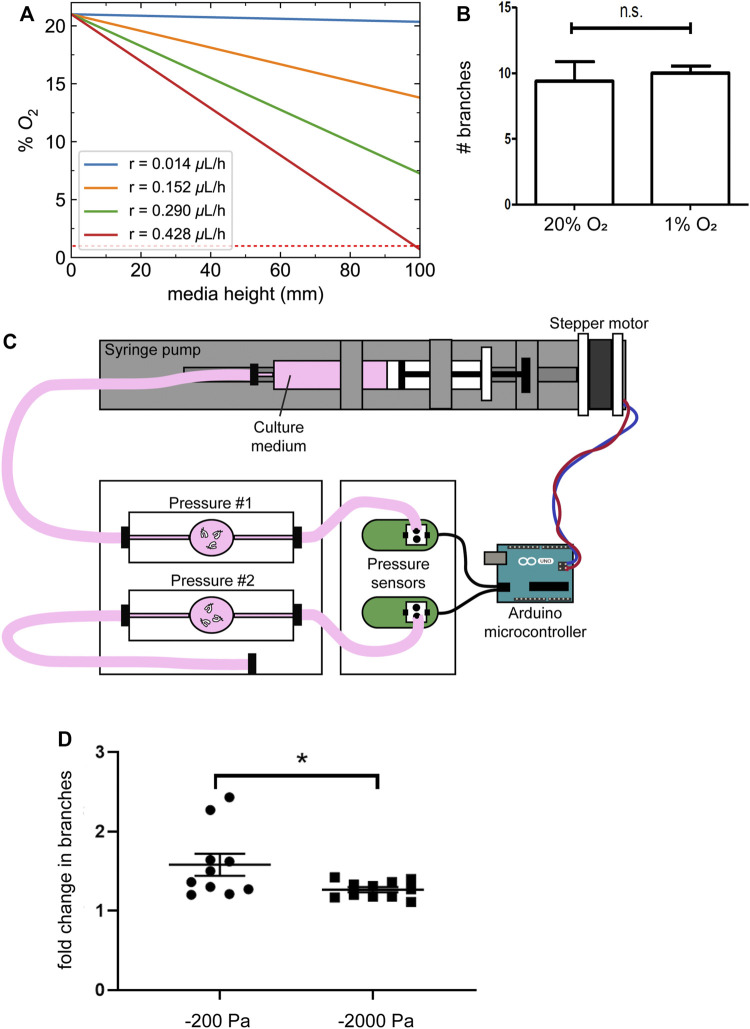
Changes in oxygen tension do not account for the effects of negative transpulmonary pressure on airway branching. **(A)** Calculated oxygen concentrations as a function of depth within the column of media above the explants, at different magnitudes of oxygen consumption rate (r). Dotted red line indicates 1% O_2_. **(B)** Number of branches formed after 24 h of culture of lung explants under normoxic or hypoxic conditions. **(C)** Schematic of culture device used to apply transpulmonary pressure in a constant volume, constant height setting. **(D)** Fold-change in the number of branches in lung explants cultured for 24 h under the conditions shown in **(C)**. Scale bars, 250 µm.

In addition to altering the magnitude of the pressure exerted on the lung explants, changing the height of the culture medium would be expected to alter diffusion of oxygen from the surface of the pressure head to the substratum (Figure 4A), with higher negative pressures corresponding to lower oxygen tensions. Fetal organs develop in a relatively hypoxic environment *in utero*, and previous studies have concluded that decreasing oxygen concentration (to 3% oxygen) results in a qualitative increase in branching morphogenesis of mouse lung explants in culture, as compared to 20% oxygen (Gebb and Jones, 2003; Van Tuyl et al., 2005), the opposite of what we observe in response to negative pressure. Nonetheless, to determine whether the most extreme changes in oxygen concentration around the lung lead to a decrease in branching morphogenesis, we cultured explants under the same volume of medium under either normoxic (20% oxygen) or hypoxic (1% oxygen) conditions. We found that culture under hypoxia does not decrease the formation of new branches in the embryonic mouse lung (Figure 4B), indicating that differences in oxygen tension cannot account for the effects of negative pressure on branching morphogenesis.

As an alternative approach, we coupled a microcontroller to a pressure sensor and a syringe pump to vary the pressure exerted on lung explants without changing the height of the medium (Figure 4C). Similar to the results obtained using the simpler culture models described above, we found that culture under increasing magnitudes of negative pressure decreased branching morphogenesis in the lung explants (Figure 4D). Taken together, these data suggest that negative transpulmonary pressure decreases airway epithelial branching, independently of dissolved solutes or oxygen transport.

### Negative Transpulmonary Pressure Decreases Airway Radius

**FIGURE 5:**
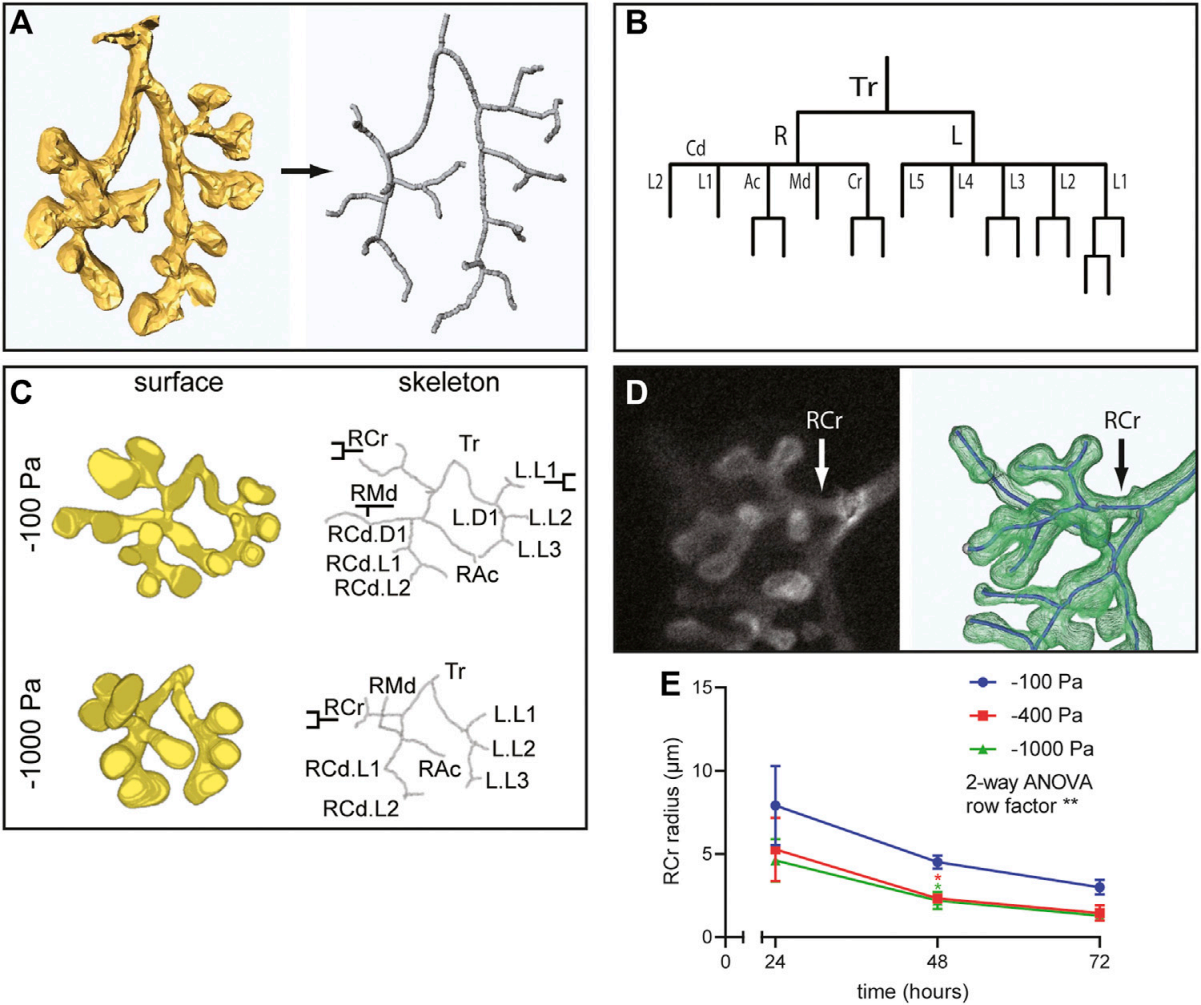
Airway lineage analysis and quantitative morphometric analysis reveal that negative transpulmonary pressure decreases branching without affecting basal epithelial surface area. **(A)** Schematic of skeletonization routine to identify branching architecture in an unbiased fashion from 3D solid models of the airways. **(B)** Airway lineage diagram of skeletonized epithelial tree depicted in **(A)**. The trachea (Tr) bifurcates into the right (R) and left (L) primary bronchi. The right primary bronchus forms the caudal (Cd), accessory (Ac), medial (Md), and cranial (Cr) branches, which generate each of the corresponding lobes of the right lung. The left primary bronchus forms five secondary bronchi (L1-L5). **(C)** 3D solid models, skeletonizations, and annotated lineages (branch names) for lungs cultured under different pressures. **(D)** Immunofluorescence analysis for E-cadherin and schematic of skeletonized RCr branch. **(E)** Mean radius of RCr as a function of time for lung explants held at different transpulmonary pressures (N = 3 experimental replicates; 3 lung explants/group). Two-way ANOVA was used to test significant variation of radius with time (row factor *p*-value = 0.0083, denoted as **). One-way ANOVA was used to compare lungs within time points (*p*-value = 0.0409 and 0.034 for −100 Pa vs. −400 and −1,000 Pa, respectively, at 48 h, denoted as *).

In addition to alterations in the number of branches, our 3D volume renderings suggested that negative transpulmonary pressure also affects the morphology of the branches. To quantify airway morphology, we skeletonized the 3D renderings (Figure 5A) and used these skeletonizations to identify the parent and daughter branches in the airway epithelial tree (Figure 5B), following a previously published classification system (Metzger et al., 2008). This branching lineage analysis revealed that the expected parent and daughter branches formed under all pressure conditions (Figure 5C); that is, we did not detect any evidence of spurious branching. To quantify possible effects on branch morphology, we focused on the right cranial (RCr) lobe of the developing epithelial tree (Figure 5D). We found that the radius of the neck of the parental branch decreased as a function of time in culture (Figure 5E). After 24 h in culture, the radius of RCr was already smaller in lungs cultured under negative transpulmonary pressure. Intriguingly, the radius of RCr continued to decrease over time such that, after 72 h in culture, those under the highest magnitudes of negative transpulmonary pressure were ∼50% of the radius of RCr in control (−100 Pa) explants (Figure 5E). These observations suggest that increasing negative transpulmonary pressure decreases both the number and alters the morphology of the branches.

### Negative Transpulmonary Pressure Decreases the Expression of *Fgf10* and Markers of Lung Maturity

**FIGURE 6:**
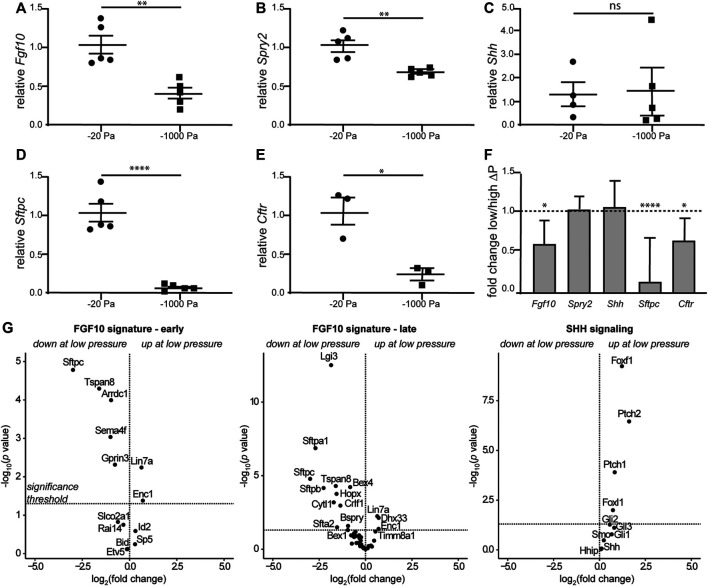
Negative transpulmonary pressure results in reduced expression of branching regulators and markers of lung maturation. **(A**–**E)** Quantitative RT-PCR analysis for the transcript levels of **(A)** *Fgf10*, **(B)** *Spry2*, **(C)** *Shh*, **(D)** *Sftpc*, and **(E)** *Cftr* after 48 h of culture. Transcript levels were normalized to those of 18S for each sample. **(F)** Relative expression of genes in lungs held under low vs. high transpulmonary pressure as assessed by bulk RNA-seq analysis; data from (Nelson et al., 2017). N = 3 experimental replicates; 5 data points/group for qRT-PCR experiments; 3 data points/group for qRT-PCR for *Cftr*. **(G)** Volcano plots for genes associated with early and late FGF10 or SHH signaling.

Airway branching morphogenesis is promoted by FGF10 *in vivo* and in culture (Bellusci et al., 1997; Min et al., 1998; Park et al., 1998; Sekine et al., 1999; De Moerlooze et al., 2000; Weaver et al., 2000; Ramasamy et al., 2007; Liu et al., 2008; Abler et al., 2009; Tang et al., 2011; Volckaert et al., 2013), and fetal conditions that result in a reduction in transpulmonary pressure and airway hypoplasia at late stages of lung development show reduced expression of *Fgf10* (Candilera et al., 2016; Wang et al., 2018). These results led us to hypothesize that although negative transpulmonary pressure exerts similar forces on the lung as positive pressure, the direction of the force differentially affects the expression of *Fgf10*. To quantify the effects of negative transpulmonary pressure on the expression of *Fgf10*, we cultured lung explants at various levels of *P*_*reservoir*_, isolated mRNA, and conducted quantitative RT-PCR analysis. We found that the levels of *Fgf10* decreased as the magnitude of negative transpulmonary pressure increased (Figure 6A), consistent with the effects of negative transpulmonary pressure on airway branching. We used a similar approach to investigate the effects of transpulmonary pressure on the principal negative regulator of FGF10 in the developing lung, SPRY2, and found that the levels of *Spry2* decreased commensurately, albeit modestly, with increasingly negative transpulmonary pressure (Figure 6B). Surprisingly, we did not observe a statistically significant difference in the expression of *Shh*, which negatively regulates *Fgf10* in the developing lung (Figure 6C). These results are consistent with observations from the nitrofen mouse model of CDH, which also shows no changes in *Shh* expression during the earliest stages of lung branching (Sato et al., 2009). We found that markers associated with lung maturation, including surfactant protein C (*Sftpc*) and cystic fibrosis transmembrane conductance regulator (*Cftr*) also decreased with increasingly negative transpulmonary pressure (Figures 6D,E). Corroborating our quantitative RT-PCR results, analysis of a previously published bulk RNA-seq dataset (Nelson et al., 2017) revealed similar trends, with *Fgf10*, *Cftr*, and *Sftpc* decreasing at more negative values of transpulmonary pressure, and no statistically significant differences in the expression of *Shh* (Figure 6F). Consistently, low transpulmonary pressure decreases the expression of the majority of FGF10-signature genes (Jones et al., 2018) while affecting only a minor subset of SHH signaling components (Figure 6G). These observations suggest that negative transpulmonary pressure disrupts airway epithelial branching and delays lung maturation, possibly in part through effects on signaling downstream of FGF10.

**FIGURE 7:**
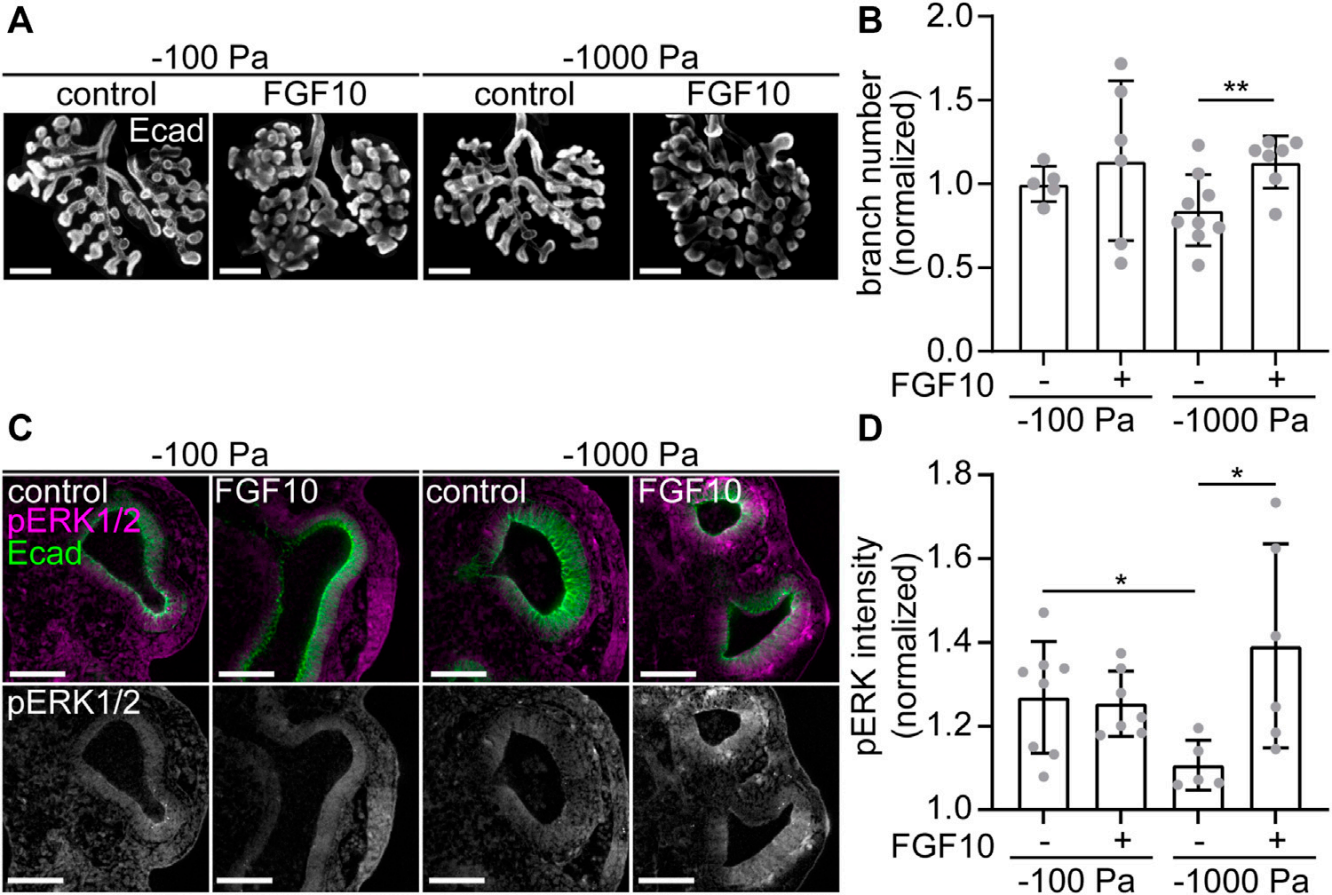
Treatment with FGF10 rescues branching of explants held under high negative transpulmonary pressure. **(A)** *E*12.5 lungs cultured under negative transpulmonary pressure with or without exogenous FGF10 for 24 h and then immunostained for E-cadherin (Ecad). Scale bars represent 250 μm. **(B)** Fold change of terminal branch number relative to controls (no FGF10, −100 Pa) in explants after 24 h of culture under different negative transpulmonary pressures, in the presence or absence of exogenous FGF10. N = 3 experimental replicates. Group compared by Student’s *t*-test. ** indicates *p* < 0.01. **(C)** *E*12.5 lungs cultured under negative transpulmonary pressure with or without exogenous FGF10 for 24 h and then immunostained for Ecad and phosphorylated ERK1/2 (pERK). Scale bars represent 50 μm. **(D)** pERK intensity normalized to background intensity in epithelial tips from lungs cultured at −100 Pa or −1,000 Pa with or without FGF10 for 24 h (*n* = 5–8, from 3 replicates). Groups were compared by *t*-test. * indicates *p* < 0.05.

If this hypothesis were correct, then we would expect that branching morphogenesis of explants cultured under the most negative transpulmonary pressure (*ΔP* = −1,000 Pa) might be rescued by supplementing the culture medium with recombinant FGF10. In the presence of vehicle control, we found that culture at *ΔP* = −1,000 Pa leads to a decrease in the number of branches, as compared to culture at *ΔP* = −100 Pa (Figures 7A,B). As predicted, treatment with exogenous FGF10 rescued this decrease in branching: explants cultured under *ΔP* = −1,000 Pa were statistically indistinguishable from those cultured at *ΔP* = −100 Pa (Figures 7A,B). Under the most negative pressure condition, exposure to FGF10 led to an increase in the activation of ERK within the epithelium, as inferred from immunofluorescence analysis (Figures 7C,D). Altogether, our data suggest that negative transpulmonary pressure suppresses branching morphogenesis in part by blocking *Fgf10* expression and downstream signaling.

## Discussion

Morphogenesis of the mammalian lung is a remarkable process. In the mouse, approximately two dozen generations of branches form recursively from a simple outpouching of foregut epithelium over a period of 10 days (Madl et al., 2010), a timeframe during which the epithelium also differentiates into several distinct cell types and begins to form the gas-exchange surface (Warburton et al., 2010). Fetal pulmonary hypoplasia is present in 15–20% of neonatal autopsies and is a frequent cause of respiratory insufficiency after birth (Harding and Hooper, 1996; Jobe, 2004; Groenman et al., 2005). Many of the molecular and genetic regulators responsible for normal airway morphogenesis have been identified (Warburton et al., 2000; Morrisey and Hogan, 2010; Ochoa-Espinosa and Affolter, 2012; Iruela-Arispe and Beitel, 2013; Swarr et al., 2019; Whitsett et al., 2019). Nonetheless, the large majority of congenital lung defects are idiopathic with respect to genetic underpinnings but are clearly accompanied by mechanical alterations in and around the fetal chest cavity (Swischuk et al., 1979; Sherer et al., 1990). Connecting these mechanical defects to their molecular effectors would suggest potential treatment strategies.

Transpulmonary pressure is normally positive during fetal lung development, with higher pressure in the luminal fluid and lower pressure in the pleural fluid (Kitterman, 1988). Pulmonary hypoplasia is observed in conditions that cause mechanical confinement of the lungs or an increase in the pressure of the pleural fluid. Our data suggest that negative transpulmonary pressure can directly inhibit branching of the airway epithelium at the earliest stages of morphogenesis in the murine lung, at least in part by blocking the expression of *Fgf10* and its downstream signaling. *Fgf10* expression in the lung is downregulated in nitrofen-induced models of CDH in mice (Acosta et al., 2001) and rats (Teramoto et al., 2003), and branching can be rescued by the exogenous addition of FGF10 in these systems (Acosta et al., 2001). These findings are consistent with those showing a positive correlation between increasing luminal pressure and the expression of *Fgf10* in lung explants (Unbekandt et al., 2008), and suggest that *Fgf10* expression is either directly or indirectly sensitive to mechanical stresses in the mouse lung. The role of FGF10 signaling in human lung development and CDH is understudied and likely has different effects than in the mouse (Danopoulos et al., 2019a; Danopoulos et al., 2019b), but this growth factor has been shown to induce the secretion of fluid into the lumen of the human fetal lung (Graeff et al., 1999) and is found at reduced levels within the amniotic fluid of fetuses with CDH (Candilera et al., 2016).

In addition to the effects on *Fgf10*, our data show that transpulmonary pressure affects the expression of several other genes associated with lung development, including *Spry2*, *Sftpc*, and *Cftr*. Consistent with our results, a recent study showed that *Sftpc* is a direct downstream target of FGF10 signaling in the pseudoglandular-stage murine lung (Isago et al., 2020), and *Sftpc* expression is also reduced in the nitrofen rat model of CDH (Guilbert et al., 2000). Also consistent with our data, exogenous FGF10 induces the expression of *Cftr* in primary airway cultures (Meyerholz et al., 2018). Based on these results, we postulate that *Cftr* is downstream of FGF10 signaling. Although we observed that the expression of *Spry2* decreased with negative transpulmonary pressure, a previous study used tracheal occlusion to increase transpulmonary pressure and inferred the opposite trend (Unbekandt et al., 2008). The underlying cause of these differences is unclear, but future studies should report the magnitudes of pressure to better enable comparisons.

Whereas our data suggest differences in downstream targets of FGF10 signaling, it is important to determine signaling upstream of *Fgf10* and to uncover how *Fgf10*-expressing cells within the lung sense their surrounding mechanical microenvironment. The mechanical regulation of *Fgf10* expression is unclear, as the growth factor is under the control of a large number of transcription factors including TBX4, FOXF1, and SP1 (Lim et al., 2002; Benjamin et al., 2010; Morrisey and Hogan, 2010). Studies have shown that YAP blocks transforming growth factor-β1 (TGFβ1)-induced SHH signaling in A549 lung epithelial cells (Isago et al., 2020), and *E*12.5-14.5 YAP-null lungs show elevated expression of *Fgf10* and decreased expression of *Shh* (Lin et al., 2017; Isago et al., 2020). It will be interesting to identify the mechanical signaling pathways that connect transpulmonary pressure to the transcriptional activation of *Fgf10*. It will also be important to determine how the cells of the embryonic lung can sense the difference between positive and negative pressures, which would be expected to generate similar magnitudes of mechanical stress but from different directions on and within the organ.

Although negative transpulmonary pressure reduces airway branching in the cultured explants, our quantitative morphometric analysis revealed no effects on the basal epithelial surface area or volume of the epithelial tree, inclusive of the lumen. Somewhat paradoxically, negative transpulmonary pressure does alter the geometry of the airways, as the radius of RCr is reduced at increasingly negative values of ΔP. However, at these more negative transpulmonary pressures, the terminal ends of the branches appeared more swollen and cystic. These data strongly suggest that branching morphogenesis (allometric growth) can be uncoupled from basal epithelial area (isometric growth) in the embryonic mouse lung, similar to observations of avian airways (Gleghorn et al., 2012; Kim et al., 2013), where the lung epithelium branches much faster than it grows. However, time-lapse experiments of longer duration are needed to determine precisely how negative transpulmonary pressure affects proliferation of the lung epithelium and the later steps of epithelial differentiation and alveolarization, which may be affected by mechanical signaling to effectors other than FGF10.

## Data Availability

The original contributions presented in the study are included in the article/Supplementary Material, further inquiries can be directed to the corresponding author.

## Abbreviations

3D, three-dimensional; BMP4, bone morphogenic protein 4; CDH, congenital diaphragmatic hernia; CFTR, cystic fibrosis transmembrane conductance regulator; *E*, embryonic day; FGF10, fibroblast growth factor 10; FGFR2, fibroblast growth factor receptor 2; MAPK, mitogen-activated protein kinase; RCr, right cranial branch; Sftpc, surfactant protein C; SHH, sonic hedgehog; SPRY4, sprouty 4; SPRY2, sprouty 2; TGFβ1, transforming growth factor-β1; YAP, yes-associated protein.
